# Hematopoietic but Not Endothelial Cell MyD88 Contributes to Host Defense during Gram-negative Pneumonia Derived Sepsis

**DOI:** 10.1371/journal.ppat.1004368

**Published:** 2014-09-25

**Authors:** Miriam H. P. van Lieshout, Adam A. Anas, Sandrine Florquin, Baidong Hou, Cornelis van't Veer, Alex F. de Vos, Tom van der Poll

**Affiliations:** 1 Center of Infection and Immunity Amsterdam, Academic Medical Center, Amsterdam, The Netherlands; 2 Center of Experimental and Molecular Medicine, Academic Medical Center, Amsterdam, the Netherlands; 3 Department of Pathology, Academic Medical Center, Amsterdam, the Netherlands; 4 Key Laboratory of Infection and Immunity, Institute of Biophysics, Chaoyang District, Beijing, China; 5 Division of Infectious Diseases, Academic Medical Center, University of Amsterdam, Amsterdam, The Netherlands; University of São Paulo, Brazil

## Abstract

*Klebsiella pneumoniae* is an important cause of sepsis. The common Toll-like receptor adapter myeloid differentiation primary response gene (MyD)88 is crucial for host defense against *Klebsiella*. Here we investigated the role of MyD88 in myeloid and endothelial cells during *Klebsiella* pneumosepsis. Mice deficient for MyD88 in myeloid (LysM-*Myd88^−/−^*) and myeloid plus endothelial (Tie2-*Myd88^−/−^*) cells showed enhanced lethality and bacterial growth. Tie2-*Myd88^−/−^* mice reconstituted with control bone marrow, representing mice with a selective MyD88 deficiency in endothelial cells, showed an unremarkable antibacterial defense. Myeloid or endothelial cell MyD88 deficiency did not impact on lung pathology or distant organ injury during late stage sepsis, while LysM-*Myd88^−/−^* mice demonstrated a strongly attenuated inflammatory response in the airways early after infection. These data suggest that myeloid but not endothelial MyD88 is important for host defense during gram-negative pneumonia derived sepsis.

## Author Summary

*Klebsiella pneumoniae* is an important causative pathogen in hospital acquired or health care associated pneumonia and sepsis. Toll-like receptors recognize conserved motifs expressed by pathogens and thereby initiate the innate immune response. Myeloid differentiation primary response gene (MyD)88 is a common adapter for multiple Toll-like receptors that is important for protective immunity during *Klebsiella* infections. The contribution of different cell types to MyD88 mediated protection is not known. We used a model of *Klebsiella* pneumonia and secondary sepsis in mice that were selectively deficient for MyD88 in specific cell-types that are implicated to be important for host defense mechanisms by use of a tissue specific gene recombination system and bone marrow transfer. We demonstrate that MyD88 in myeloid cells, but not in endothelial cells, is important for host defense during pneumonia derived sepsis caused by *Klebsiella pneumoniae*.

## Introduction

Globally, lower respiratory tract infections are in the top ten causes of death, both in high- and low-income countries [1]. Pneumonia is the most common cause of sepsis and frequently caused by gram-negative pathogens from the family of Enterobacteriaceae, including *Klebsiella (K.) pneumoniae* [2]–[4]. Increasing rates of extended-spectrum β-lactamases producing Enterobacteriaceae are a major health concern and make the development of new therapies urgent, since infection with such pathogens is associated with increased mortality [5]–[7].

Infection is detected by sensors of the innate immune system collectively called pattern recognition receptors [8], [9]. Toll-like receptors (TLRs) prominently feature herein, able to detect a variety of conserved microbial patterns as well as “danger signals” released from host cells as a consequence of injurious inflammation. As such, TLRs play an important role in the initiation and amplification of the host response [8], [9]. The universal adaptor for all TLRs except TLR3 is myeloid differentiation primary response gene (MyD)88, that propagates the signal of activated TLRs intracellularly, leading to NFκB and MAP kinase activation. In addition, MyD88 mediates IL-1β and IL-18 receptor signaling [10]. We and others recently demonstrated the importance of MyD88 dependent signaling for survival and antibacterial defense during *K. pneumoniae* infection [3], [11], [12]. During respiratory tract infection different MyD88 expressing cells may contribute to host defense, including innate immune cells, such as alveolar macrophages, intraepithelial dendritic cells and migrated leukocytes, and parenchymal cells, such as lung epithelium and endothelium [13]–[15]. By creating chimeric mice using bone marrow (BM) transplantation, we reported the importance of MyD88 in both radiosensitive (hematopoietic) cells and radioresistant (parenchymal) cells for antibacterial defense and survival during *Klebsiella* pneumonia derived sepsis [12].

Whereas the role of hematopoietic cells in host defense against bacteria is undisputed, there are only few reports about the specific contribution of the vascular endothelium to the pathophysiology of infection and sepsis. Some evidence points to an attenuation of tissue and organ injury during polymicrobial sepsis when endothelial NFκB signaling was specifically targeted, without an effect on bacterial clearance [16]–[19]. However on the other hand the specific expression of endothelial TLR4 was reported to be sufficient for adequate bacterial clearance in a model of gram-negative infection [20]. Therefore, we here aimed to study the role of MyD88 dependent signaling in myeloid and endothelial cells during *K. pneumoniae* pneumosepsis by using mice with cell-specific targeted deletion of *Myd88* and BM transfer. We demonstrate that myeloid, but nor endothelial cell MyD88 is important for host defense during pneumonia derived sepsis caused by *Klebsiella*.

## Results

### Genetic and functional characterization of primary cells from *LysM-Myd88^−/−^* and *Tie2-Myd88^−/−^* mice

**Figure 1:**
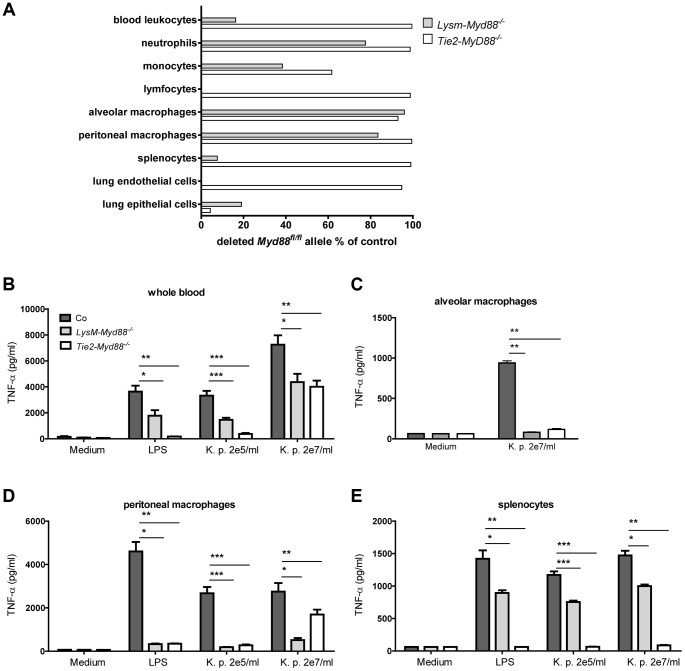
Genetic and functional characterization of primary cells from LysM-*Myd88^−/−^* and Tie2-*Myd88^−/−^* mice. The residual amount of the MyD88^fl/fl^ allel in blood and primary cells LysM-*Myd88^−/−^* and Tek-*Myd88^−/−^* mice was quantified via qRT-PCR relative to the unaffected *Socs-3* gene. The amount of remaining “floxed” MyD88 region in LysM-MyD88^−/−^ and Tek-MyD88^−/−^ mice was calculated using the 2^-deltaCt^ (ΔΔCt) method using the amount of genomic DNA from *Myd88^fl/fl^* mice for the no-deletion control. The deletion efficiency was calculated as (1 - residual Myd88^fl^) ×100% **(A)**. Whole blood **(B)**, alveolar and peritoneal macrophages **(C,D)** and splenocytes **(E)** derived from control, LysM-*Myd88^−/−^* and Tie2-*Myd88^−/−^* mice (n = 3 per group) were in vitro stimulated with LPS derived from *Klebsiella pneumoniae* (1 µg/ml) or heat killed *K. pneumoniae* in two concentrations (2×10^5^ CFU/ml or 2×10^7^/ml) for 20 hours. Data are expressed as mean (SE). * *p*<0.05, ** *p*<0.01, *** *p*<0.001.

To investigate the relative contribution of MyD88 dependent signaling in myeloid and endothelial cells to protective immunity during gram-negative pneumosepsis we crossed mice homozygous for the conditional *Myd88* flox allele (*Myd88^fl/fl^* mice) [21] with mice expressing Cre under control of the myeloid cell LysM promoter (to generate LysM-*Myd88^−/−^* mice) [22] or the myeloid plus endothelial cell Tie2 promoter (to generate Tie2-*Myd88^−/−^* mice) [23]. To determine the efficiency of Cre-induced *Myd88* deletion in specific cell types, we performed qPCR to quantify the remaining *Myd88^fl/fl^* in blood total leukocytes, granulocytes, monocytes and lymphocytes, in alveolar and peritoneal macrophages, in splenocytes and in lung endothelial and epithelial cells (figure 1A). As expected, the deletion efficiency of Cre in LysM-*Myd88^−/−^* was very high in the myeloid compartment, especially in macrophages, granulocytes and to a lesser extent monocytes; lymphocytes and endothelial cells were unaffected. As anticipated, the *Myd88^fl/fl^* allele was almost completely absent in endothelial cells of Tie2-*Myd88^−/−^* mice. In addition, excision of the *Myd88^fl/fl^* allele was also virtually complete in all hematopoietic cell types of Tie2-*Myd88^−/−^* mice, as well as in lymphocytes and (accordingly) in splenocytes. Next, to determine the functional consequences of these Cre-mediated cell-specific *Myd88* deletions, we incubated whole blood leukocytes, alveolar and peritoneal macrophages and splenocytes obtained from LysM-*Myd88^−/−^*, Tie2-*Myd88^−/−^* and control mice with *K. pneumoniae* LPS or heat-killed *K. pneumoniae*, using TNFα release as readout; we focused on these cell types since they confer protective functions during infection and sepsis [24]–[26]. In agreement with the genetic characterization of cells from LysM-*Myd88^−/−^* and Tie2-*Myd88^−/−^* mice, whole blood leukocytes from both genotypes showed a clearly reduced responsiveness to *Klebsiella* and *Klebsiella* LPS, with Tie2-*Myd88^−/−^* leukocytes showing the largest defect (figure 1B). In addition, LysM-*Myd88^−/−^* and Tie2-*Myd88^−/−^* alveolar and peritoneal macrophages displayed strongly reduced TNFα release upon stimulation (figure 1C,D), while the strongest defect in splenocyte responsiveness was seen in Tie2-*Myd88^−/−^* cell cultures (figure 1E). Together these results indicate that Tie2-*Myd88^−/−^* mice are MyD88 deficient in hematopoietic, lymphoid and endothelial cells, while in LysM-*Myd88^−/−^* mice MyD88 deficiency is restricted to hematopoietic cells.

### *LysM-Myd88^−/−^* and *Tie2-Myd88^−/−^* mice demonstrate a strongly impaired host defense during gram-negative pneumosepsis

**Figure 2:**
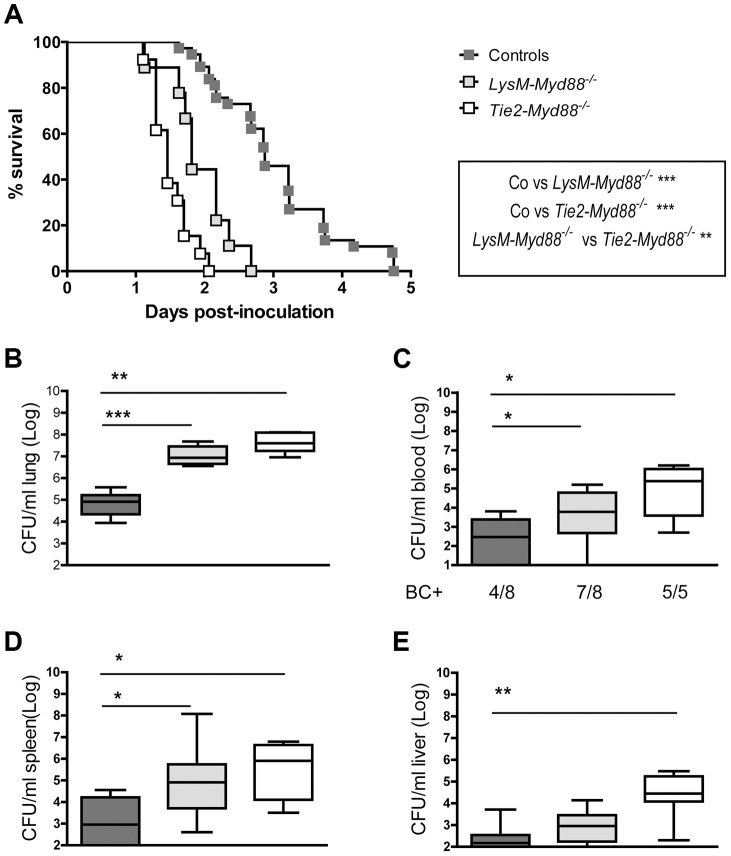
Impaired survival and bacterial defense in LysM-*Myd88^−/−^* and Tie2-*Myd88^−/−^* mice. Control, LysM-*Myd88^−/−^* and Tie2-*Myd88^−/−^* mice were intranasally infected with ∼6×10^3^ CFU *K. pneumoniae*. Survival of control (dark grey symbols, n = 37), LysM-*Myd88^−/−^* (light grey symbols, n = 9) and Tie2-*Myd88^−/−^* mice (white symbols, n = 13) expressed as Kaplan-Meier plot **(A)**, bacterial loads in lung **(B)**, blood **(C)**, spleen **(D)** and liver **(E)**, of control (dark grey bars, n = 8), LysM-*Myd88^−/−^* (light grey bars, n = 8) and Tie2-*Myd88^−/−^* mice (white bars, n = 5 mice). Data are expressed as box-and-whisker diagrams depicting the smallest observation, lower quartile, median, upper quartile, and largest observation. BC+ = number of positive blood cultures. Survival curves were compared with Log-Rank test Bacterial loads were compared to control mice determined with Mann-Whitney *U* test: * *p*<0.05, ** *p*<0.01, *** *p*<0.001.

Next, we infected LysM-*Myd88^−/−^*, Tie2-*Myd88^−/−^* and *Myd88^fl/fl^* Cre negative control mice with *K. pneumoniae* via the airways and monitored mortality during a 5-day follow up (figure 2A). LysM-*Myd88^−/−^* and Tie2*-Myd88^−/−^* mice displayed massive mortality within the first 2 days after infection with median survival times of 1.8 and 1.5 days respectively, while control mice had a median survival time of 2.9 days (both *p*<0.001 versus control mice). Notably, Tie2-*Myd88^−/−^* mice showed an accelerated mortality relative to LysM-*Myd88^−/−^* mice (*p*<0.01 for the difference between groups). To obtain insight in the cause of early lethality of LysM-*Myd88^−/−^* and Tie2-*Myd88^−/−^* mice we next infected mice with *Klebsiella* in a separate experiment and harvested lungs, blood, spleen and liver for quantitative cultures 24 hours post infection (*i.e.* shortly before the first deaths were expected to occur), seeking to collect data representative for host defense at the primary site of infection and bacterial dissemination. At this time point, both LysM-*Myd88^−/−^* and Tie2-*Myd88^−/−^* mice had ≥2-log more bacteria in their lungs relative to control mice (*p*<0.01 and 0.001 respectively compared to controls, figure 2B). Moreover, bacterial counts were significantly higher in blood and spleen of LysM-*Myd88^−/−^* and Tie2-*Myd88^−/−^* mice (both *p*<0.05 compared to control mice, figure 2C and D). In addition, Tie2-*Myd88^−/−^* mice had significantly higher amounts of bacteria in their livers (*p*<0.01 compared to control mice, figure 2E). Tie2-*Myd88^−/−^* mice had higher bacterial counts when compared with LysM-*Myd88^−/−^* mice in all body sites, although these differences did not reach statistical significance. Together these data indicate that LysM-*Myd88^−/−^* and Tie2-*Myd88^−/−^* mice demonstrate a strongly enhanced bacterial growth and dissemination during gram-negative pneumonia derived sepsis, resulting in accelerated mortality.

### *LysM-Myd88^−/−^* and *Tie2-Myd88^−/−^* mice show modest alterations in the inflammatory and injurious response during gram-negative pneumosepsis

**Figure 3:**
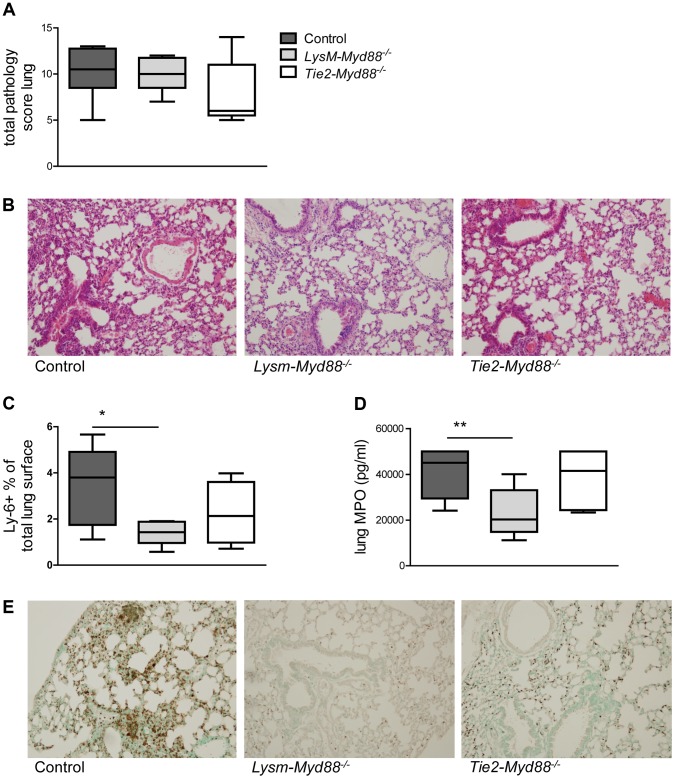
Lung inflammatory response. Mice were intranasally infected with ∼6×10^3^ CFU *K. pneumoniae*; Histological scores 24 hours after infection determined as described in the Methods section, in control (dark grey, n = 8), LysM-*Myd88^−/−^* (light grey, n = 8) and Tie2-*Myd88^−/−^* mice (white, n = 5) **(A)**. Panel **(B)** shows representative lung histology of control, LysM-*Myd88^−/−^* and Tie2-*Myd88^−/−^* mice H&E staining, original magnification 20×. Neutrophil influx compared between mouse groups as reflected by Ly6 lung surface positivity **(C)** and whole lung MPO levels **(D)**. Panel **(E)** shows representative images of Ly-6 staining on lung slides from control, LysM-*Myd88^−/−^* and Tie2-*Myd88^−/−^* mice; Data are expressed as box-and-whisker diagrams depicting the smallest observation, lower quartile, median, upper quartile, and largest observation. * *p*<0.05, ** *p*<0.01.

To obtain insight in local inflammation at the primary site of infection we harvested lungs from LysM-*Myd88^−/−^*, Tie2-*Myd88^−/−^* and control mice 24 hours post infection for semi-quantitative histopathology, focusing on key histological features characteristic for severe pneumonia (figure 3). The extent of lung pathology did not differ between groups. LysM-*Myd88^−/−^* mice had lower myeloperoxidase (MPO) concentrations in whole lung homogenates, indicative of a reduced neutrophil content. In accordance, the number of Ly6+ cells was lower in LysM-*Myd88^−/−^* mice relative to controls. To obtain further insight in the role of MyD88 in cells targeted by LysM- and Tie2-driven Cre recombinase in lung inflammation during *Klebsiella* pneumonia, we measured the levels of the proinflammatory cytokines IL-1β, TNF-α, IL-6, the anti-inflammatory cytokine IL-10 and the neutrophil attracting chemokines CXCL-1 and CXCL-2 in lung homogenates (table 1). The pulmonary concentrations of all mediators were similar in LysM-*Myd88^−/−^*, Tie2-*Myd88^−/−^* and control mice, with the exception of TNF-α levels which were significantly lower in Tie2-*Myd88^−/−^* mice (*p*<0.01 compared to controls). Plasma IL-6 levels were significantly increased in LysM-*Myd88^−/−^* and Tie2-*Myd88^−/−^* mice (*p*<0.05 to 0.01 respectively compared to controls, table 1) likely as a result of higher bacterial loads. In addition, we determined E-selectin levels in both lung homogenates and plasma as a reflection of endothelial cell activation [27] and observed that lung levels of E-selectin were significantly increased in Tie2-*Myd88^−/−^* mice, probably as a result of the higher bacterial burden (*p*<0.05 compared to control mice) (figure S1).

**Table 1:** Inflammatory response in *LysM-MyD88^−/−^* and *Tie2-MyD88^−/−^* during *K. pneumonia* pulmonary tract infection.

	Co	*LysM-MyD88^−/−^*	*Tie2-MyD88^−/−^*
***Lung***			
**TNF-α**	2632 (295)	2181 (204)	1610 (159)**
**IL-1β**	3523 (410)	2989 (341)	2559 (350)
**IL-6**	3417 (587)	5112 (697)	4798 (1666)
**IL-10**	bd	bd	bd
**CXCL-1**	3502 (855)	5083 (1091)	5675 (1479)
**CXCL-2**	28270 (4621)	37871 (6138)	27254 (8982)
***Plasma***			
**TNF-α**	12 (4)	85 (41)	55 (20)*
**IL-6**	240 (47)	2364 (1251)*	783 (96)**
**IL-10**	bd	bd	bd
**CCL-2**	468 (330)	141 (48)	1297 (428)*

Control, LysM-MyD88^−/−^ and Tie2-MyD88^−/−^ mice were inoculated with ∼6×10^3^ CFU *K. pneumoniae* and sacrificed 24 hours later. Homogenates were prepared from right lungs. Cytokine and chemokine levels are presented in pg/ml lung homogenate or plasma. Data are mean (SE) of 5–8 mice per group.

* p<0.05,

** *p*<0.01 vs control mice.

Bd = below detection.

The model of *Klebsiella* pneumonia and sepsis used here is associated with rises in the plasma concentrations of LDH (indicative for cellular injury in general) and AST (reflecting hepatocellular injury) in the late stage of infection [28]. To study if the absence of MyD88 in myeloid and/or endothelial cells affected the degree of liver and cellular injury we determined the plasma levels of these parameters but observed no differences (figure S2).

Together these data suggest that the increased mortality in LysM-*Myd88^−/−^* and Tie2-*Myd88^−/−^* mice occurred as a result of overwhelming bacterial growth rather than as a result of pulmonary or distant organ injury.

### MyD88 dependent signaling in the hematopoietic compartment is crucial for antibacterial defense while MyD88 in endothelial cells is not important

**Figure 4:**
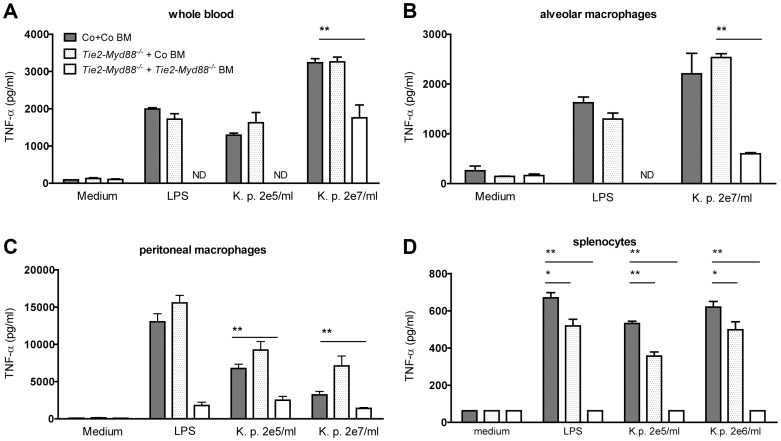
Bone marrow transfer restores responsiveness of hematopoietic cells from Tie2-*Myd88^−/−^* mice to *Klebsiella*. Whole blood **(A)**, alveolar and peritoneal macrophages **(B,C)** and splenocytes **(D)** derived from control mice transplanted with control bone marrow (Co+ Co BM, grey bars) and Tie2-*Myd88^−/−^* mice transplanted with control bone marrow (Tie2-*Myd88^−/−^*+Co BM, white dotted bars) or Tie2-*Myd88^−/−^* bone marrow (Tie2-*Myd88^−/−^*+Tie2-*Myd88^−/−^* BM, white bars). (n = 2–3 per group) were in vitro stimulated with LPS derived from *Klebsiella pneumoniae* (1 µg/ml) or heat killed *K. pneumoniae* in two concentrations (2×10^5^ CFU/ml or 2×10^7^/ml) for 20 hours. Data are expressed as mean (SE). * *p*<0.05, ** *p*<0.01. ND = not determined.

**Figure 5:**
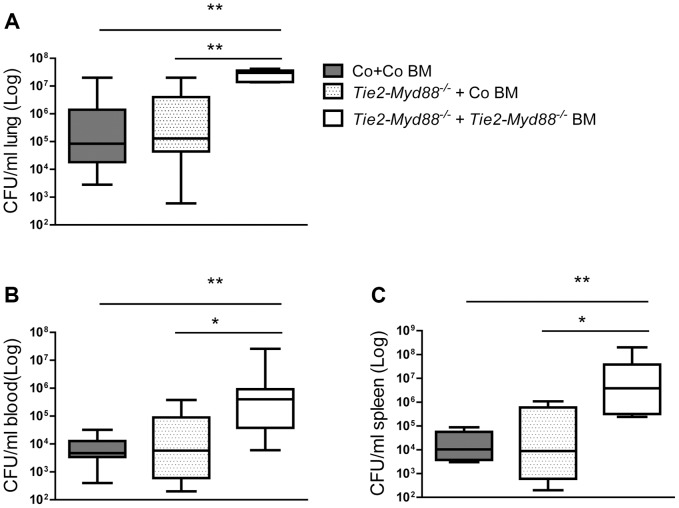
The absence of MyD88 in the hematopoietic compartment determines the impaired antibacterial defense of Tie2-*Myd88^−/−^* mice. Control and Tie2-*Myd88^−/−^* mice were irradiated and injected with control or Tie2-*Myd88^−/−^* bone marrow cells. Six weeks after transplantation, mice were infected with 6×10^3^ CFU *K. pneumoniae* and sacrificed after 24 hours. Bacterial loads in lung **(A)**, blood **(B)**, spleen **(C)** of control mice transplanted with control bone marrow (Co+ Co BM, grey bars, n = 8) and Tie2-*Myd88^−/−^* mice transplanted with control bone marrow (Tie2-*Myd88^−/−^*+Co BM, white dotted bars) or Tie2-*Myd88^−/−^* bone marrow (Tie2-*Myd88^−/−^*+Tie2-*Myd88^−/−^* BM, white bars). Data are expressed as box-and-whisker diagrams depicting the smallest observation, lower quartile, median, upper quartile, and largest observation. * *p*<0.05, ** *p*<0.01.

Considering that Tie2-*Myd88^−/−^* mice have strongly impaired MyD88 signaling in hematopoietic and endothelial cells, we decided to restore the hematopoietic compartment of Tie2-*Myd88^−/−^* mice with BM of *Myd88^fl/fl^* control mice after lethal irradiation, thereby creating mice with a more exclusive MyD88 deficiency in endothelial cells. In order to adequately estimate the effect size, we created two control groups: Tie2-*Myd88^−/−^* mice transplanted with Tie2-*Myd88^−/−^* BM and control mice transplanted with control BM. After 6 weeks of recovery, we infected mice with *K. pneumoniae* intranasally and sacrificed them 24 hours later. In addition, to check the efficiency of the BM transplantation to restore the responsiveness of relevant cell types from Tie2-*Myd88^−/−^* mice to *Klebsiella*, we euthanized 2–3 uninfected mice of each recipient group and repeated cell stimulation experiments as described above. These experiments revealed that transfer of control BM in Tie2-*Myd88^−/−^* mice fully restored the capacity of blood leukocytes, and alveolar and peritoneal macrophages, and partially that of splenocytes, to produce TNFα upon exposure to *Klebsiella in vitro* (figure 4A–D). The response of cells obtained from the two control groups transplanted with isogenic BM (control mice+control BM and Tie2-*Myd88^−/−^* mice+Tie2-*Myd88^−/−^* BM) replicated the impaired response of untransplanted Tie2-*Myd88^−/−^* mice relative to control mice. Importantly, after 24 hours of infection, lung bacterial loads of Tie2-*Myd88^−/−^* +control BM mice were indistinguishable from control+control BM mice, while the difference between Tie2-*Myd88^−/−^*+Tie2-*Myd88^−/−^* BM mice and control+control BM mice phenocopied the difference between Tie2-*Myd88^−/−^* and control mice observed in untransplanted mice (*p*<0.001, figure 5A). In line, lung bacterial levels were significantly lower in Tie2-*Myd88^−/−^*+control BM mice compared to Tie2-*Myd88^−/−^*+Tie2-*Myd88^−/−^* BM mice (*p*<0.01). Bacterial numbers in blood and spleen confirmed the protective effect of reconstitution of the hematopoietic compartment of Tie2-*Myd88^−/−^* mice with MyD88 sufficient BM (*p*<0.05 versus Tie2-*Myd88^−/−^*+Tie2-*Myd88^−/−^* BM mice, figure 5B,C). The extent of lung pathology, lung MPO levels and the number of Ly6+ cells in lung tissue were not different between groups (figure S3). Moreover, lung and plasma cytokine/chemokine and E selectin concentrations were not affected by the selective absence of endothelial MyD88 in Tie2-*Myd88^−/−^*+control BM mice, except for slightly lower lung levels of IL-10 compared to control+control BM mice (table S1; figure S4)). Also, the plasma levels of AST and LDH did not differ between groups (figure S4). Together, these data indicate that endothelial cell MyD88 has no role in antibacterial defense or in lung or distant organ injury after infection with *Klebsiella* via the airways.

### *LysM-Myd88^−/−^* mice demonstrate an attenuated early inflammatory response

**Figure 6:**
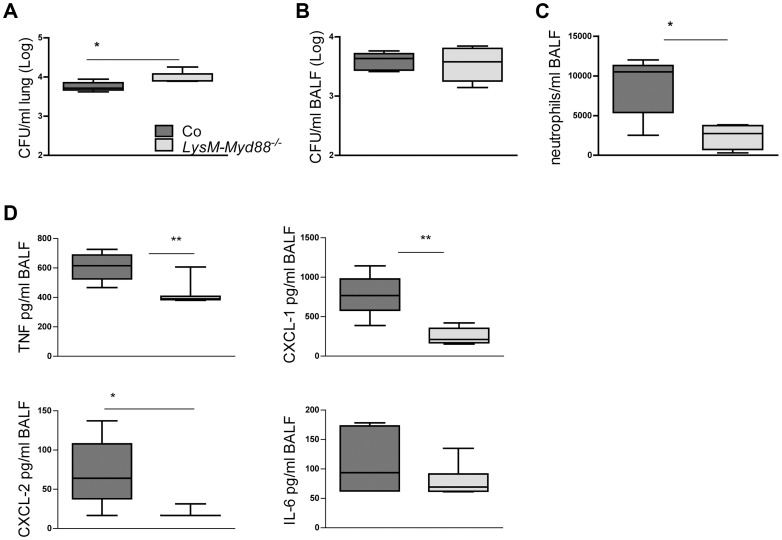
*LysM-Myd88^−/−^* mice demonstrate an attenuated early inflammatory response. Control and LysM-*Myd88^−/−^* mice were intranasally infected with ∼6×10^3^ CFU *K. pneumoniae*. Bacterial loads in lung **(A)** and BALF **(B)**, number of neutrophils **(C)** and levels of TNF-α, CXCL-1, CXCL-2 and IL-6 **(D)** in BALF of control (dark grey symbols, n = 8) and LysM-*Myd88^−/−^* mice (light grey symbols, n = 8). Data are expressed as box-and-whisker diagrams depicting the smallest observation, lower quartile, median, upper quartile, and largest observation. * *p*<0.05, ** *p*<0.01.

Mice with a complete MyD88 deficiency show a strongly impaired antibacterial defense after infection with *Klebsiella* via the airways caused by a mitigated neutrophil recruitment into the airways associated with strongly reduced local levels of neutrophil attracting mediators [11]. We wished to determine whether a similar mechanism is at play in LysM-*Myd88^−/−^* mice. Thus, LysM-*Myd88^−/−^* and control mice were infected with *K. pneumoniae* intranasally and lungs and bronchoalveolar lavage (BAL) fluid was harvested 6 hours later. LysM-*Myd88^−/−^* mice showed higher bacterial loads in whole lung homogenates, but not in BAL fluid (figure 6A). Importantly, LysM-*Myd88^−/−^* mice displayed a strongly attenuated influx of neutrophils into the bronchoalveolar compartment (figure 6B), which was associated with markedly reduced levels of TNFα, CXCL-1 and CXCL-2 in BAL fluid; IL-6 concentrations in BAL fluid did not differ between groups (figure 6D). Hence, these data suggest that LysM-*Myd88^−/−^* mice replicate the phenotype of *Myd88^−/−^* mice with regard to impaired neutrophil influx in the airways during early *Klebsiella* pneumonia at least in part caused by a reduced chemotactic gradient due to impaired chemoattractant production.

## Discussion

Several MyD88 dependent TLRs are known to be important for the innate immune response to respiratory tract infection with *K. pneumoniae*, particularly TLR4 and TLR9, and during late stage infection or in the presence of high bacterial numbers, TLR2 [29]–[32]. Since TLRs and other innate immune sensors are widely distributed among different cell types in the airways, comprising both hematopoietic and non-hematopoietic cells, our laboratory engaged in several studies seeking to dissect the cell-specific contribution of TLR and MyD88 signaling in host defense during *Klebsiella* pneumonia derived sepsis [12], [32]. Using BM chimeras we reported that TLR2 and TLR4 expression in hematopoietic cells are crucial for antibacterial defense, while MyD88 in hematopoietic and parenchymal cells is equally important [12], [32]. BM transplantation can introduce artefacts caused by the irradiation and/or incomplete replacement of recipient hematopoietic cells, and cannot provide detailed information about the specific cell type that is affected [33]. In the present study we used the Cre-lox system combined with BM transfer to study the role of myeloid and endothelial cell specific MyD88 signalling in the host response during *Klebsiella* induced pneumosepsis. We demonstrate that while myeloid MyD88 contributes significantly to host defense, endothelial cell MyD88 has no role herein.

Endothelial cells are resident cells implicated in sepsis pathogenesis and the induction of organ injury [34]. Earlier investigations examined the contribution of TLR and NFκB signaling within the vascular endothelium to the host response during experimental sepsis. Inhibition of endothelial NFκB signaling by overexpression of a degradation-resistant form of the NF-κB inhibitor I-κBα under the control of the endothelial cell specific VE-cadherin-5 promoter attenuated tissue inflammation and organ injury during endotoxemia and abdominal sepsis [16]–[19]. In addition, these mice displayed strongly reduced coagulation activation upon administration of endotoxin [19]. Endothelial cell specific NFκB inhibition did not influence the clearance of *Listeria monocytogenesis*, *Streptococcus pneumoniae* or *Salmonella enterica* after intravenous infection [16]–[19]. However, transgenic Tie2 driven expression of TLR4 in *Tlr4^−/−^* mice, resulting in mice with TLR4 expression restricted to endothelial cells, was sufficient for adequate bacterial clearance after intraperitoneal infection with *Escherichia coli* [20]. We used mice with Tie2 driven expression of Cre recombinase to delete hematopoietic and endothelial MyD88 in *Myd88* ^fl/fl^ mice and observed a strongly impaired host defense as reflected by very high bacterial loads and increased mortality. Previous studies support Tie2 expression in hematopoietic cells and the lack of specificity for endothelial cells [23], [35]. Similarly, the VE-cadherin-5 promoter is reported to drive Cre recombinase gene expression not only in endothelial cells but also in a subset of hematopoietic cells [36]. As such, the Cre-lox system seems less suitable to specifically study the function of genes in endothelial cells. Therefore, to generate mice with endothelial cell specific MyD88 deficiency, we reconstituted Tie2-*Myd88^−/−^* mice with BM of control mice and confirmed functional recovery of their hematopoietic cells with regard to responsiveness to *Klebsiella*. These mice were indistinguishable from control mice with regard to antibacterial defense, inflammation and distant organ injury, strongly suggesting that endothelial cell MyD88 does not play an important role in the host response during *Klebsiella* induced pneumosepsis. Although this “negative” finding may seem to contrast with previous studies on the role of endothelial cells in severe infection [16]–[20], our approach clearly differs from these earlier investigations, both with regard to the target of genetic manipulation (deletion of MyD88 versus inhibition of NFκB [16]–[19] and endothelial cell TLR4 expression on an otherwise TLR4 deficient background [20]) and the sepsis model used (pneumonia versus abdominal or intravenous infection [16]–[20]). Importantly, mice with TLR4 exclusively on endothelial cells were unable to recruit neutrophils into the lungs upon intratracheal LPS administration [20] and, similarly, studies in TLR4 BM chimeras have indicated that neutrophil influx after airway exposure to LPS occurs by mechanisms that do not rely on TLR4 expression by radioresistant (including endothelial) cells [37], which is completely consistent with our present data. Of note, findings in TLR4 BM chimeras have suggested that neutrophil accumulation in lungs upon intravenous LPS challenge does largely dependent on TLR4 in radioresistant cells [38], indicating that the role of cell-specific TLR signaling in neutrophil recruitment likely depends on the route by which the bacterial stimulus is administered.

*LysM*-Cre mediated deletion of the floxed *Myd88* allele resulted in MyD88 deficiency especially in macrophages and neutrophils, and to a lesser extent monocytes [22], [39]. Clearly, these myeloid cell MyD88 deficient mice showed a strongly compromised host defense after infection with *Klebsiella*, as reflected by enhanced mortality, increased bacterial numbers at the primary site of infection and an impaired early neutrophil influx and cytokine/chemokine release in the airways. Thus, MyD88 expressed by alveolar macrophages and neutrophils is essential for initiation of an adequate early innate immune response in the lung after infection with *Klebsiella* via the airways and the absence thereof results in uncontrolled bacterial growth and death. The phenotype of LysM-*Myd88* ^−/−^ mice was very similar to the previously documented phenotype of *Myd88^−/−^* mice during *Klebsiella* pneumonia [3], [11], [12], underlining the importance of myeloid cell MyD88 during respiratory tract infection. The *Klebsiella* strain used here cannot be killed by macrophages or neutrophils *in vitro*, illustrating its high virulence and precluding analysis of a possible direct role of MyD88 in killing. Previous studies have reported a role for MyD88 in killing of commensal and attenuated pathogenic Gram-negative bacteria [40], but not in killing of *Listeria* by macrophages [41].

While innate immunity is important for antibacterial defense, it can also cause harm by hyperinflammation induced organ injury [42], [43]. Deficiency of MyD88 has been shown to be protective in polymicrobial sepsis, in which especially liver injury was found to be associated with MyD88 dependent signaling [43], [44]. A recent study demonstrated that mice with selective expression of MyD88 in myeloid cells displayed enhanced hepatocellular injury during abdominal sepsis induced by cecal ligation and puncture [45]. Here we found no evidence for a role of either myeloid or endothelial cell MyD88 in hepatocellular damage during pneumonia derived sepsis caused by *K. pneumoniae*. Hence, although MyD88 may contribute to organ injury during sepsis, its role likely depends on the type and primary source of the infection.

Using MyD88 BM chimeras, we recently reported a role for both hematopoietic and parenchymal MyD88 in host defense in this model [12]. Since we could not demonstrate a role for endothelial cell MyD88 in the present investigation, MyD88 expressed in the respiratory epithelium may be involved. Indeed, lung epithelial cells have been implicated in host defense during respiratory tract infection [15]. The importance of MyD88 dependent signaling in lung epithelial cells was recently elegantly demonstrated in a model of *Pseudomonas* pneumonia in epithelial specific MyD88 knock-in mice [46], [47]. Selective expression of MyD88 in the airway epithelium was sufficient for neutrophil recruitment to the site of infection and bacterial clearance [47]. In addition, transgenic overexpression of IκB-α in alveolar and bronchial epithelium in mice resulted in a reduced neutrophil influx into BAL fluid upon intrapulmonary delivery of LPS [48], [49] and an increased growth of the gram-positive pathogen *Streptococcus pneumoniae* upon intratracheal infection [50]. Studies using mice in which *Myd88* is deleted specifically in respiratory epithelium are warranted to establish the role of epithelial MyD88 in host defense against *Klebsiella* pneumonia derived sepsis. However, our first preliminary results with mice generated from intercrossings of *Myd88* ^fl/fl^ mice and mice with Cre recombinase controlled by the surfactant protein C promoter [51], resulting in mice with a targeted deletion of *Myd88* in distal airway epithelium, suggest that epithelial cell MyD88 does not contribute to protective immunity during *Klebsiella* pneumonia. Therefore, our earlier data using MyD88 BM chimeras [12] may have been confounded by incomplete replacement of recipient (MyD88 sufficient) hematopoietic cells.

LysM-*Myd88^−/−^* mice showed a strongly impaired neutrophil influx into the bronchoalveolar space 6 hours after infection with *Klebsiella*, together with markedly reduced local concentrations of neutrophil attracting mediators such as TNF-α, CXCL1 and CXCL2. Notably, global MyD88 deficiency similarly results in an early impairment of neutrophil chemoattractant release and neutrophil migration into the airways in mouse models of pneumonia caused by a variety of bacterial and viral species [11], [52]–[57], as well as during sterile lung inflammation [58], [59]. These data suggest that hematopoietic and global MyD88 deficiency impair host defense during pneumonia by a largely similar mechanism that involves an inability to produce a chemotactic gradient that would normally attract neutrophils to the site of the infection. Notably, MyD88 deficient mice showed extensive lung inflammation, including high E-selectin levels, at 24 hours after infection, suggesting that these late responses can be induced by *Klebsiella* via MyD88-independent mechanisms (e.g., via the TRIF pathway) in the presence of the (by then) very high bacterial loads. Similarly, global *Myd88^−/−^* mice were previously reported to show profound lung inflammation during late stage bacterial pneumonia in the presence of high bacterial loads [54], [56], [60]. E-selectin, while implicated in the rolling of neutrophils along the vascular endothelium [61], does not seem to play a role in neutrophil recruitment to the lungs elicited by bacterial stimuli [62], [63].

In conclusion, to our knowledge, we here report for the first time on the role of MyD88 in myeloid and endothelial cells in severe bacterial infection, using a clinically relevant model of gram-negative pneumonia derived sepsis characterized by gradual growth of bacteria at the primary site of infection followed by dissemination, tissue injury and death. While myeloid MyD88 was crucial for protective immunity, endothelial MyD88 played no role herein. Our results suggest that myeloid MyD88 deficiency results in enhanced lethality during *Klebsiella* pneumonia by a mechanism that involves a strongly attenuated early inflammatory response at the primary site of infection and as a consequence thereof uncontrolled bacterial growth. These data provide new insights in the pathophysiology of gram-negative sepsis and may be helpful for the development of therapeutics aimed at specific cell types.

## Materials and Methods

### Ethics statement

Experiments were carried out in accordance with the Dutch Experiment on Animals Act and approved by the Animal Care and Use Committee of the University of Amsterdam (Permit number: DIX 100121, sub-protocols DIX102300 and DIX101613).

### Animals

Homozygous *Myd88^fl/fl^* mice [21] were crossed with LysM-Cre [22] or Tie2-Cre mice [23], both obtained from the Jackson Laboratory (Bar Harbor, Maine), to generate myeloid (LysM-*Myd88^−/−^*) and myeloid plus endothelial cell (Tie2-*Myd88^−^* ^/^) specific MyD88 deficient mice. *Myd88* ^fl/fl^ Cre negative littermates were used as controls. All mice were backrossed at least 8 times to a C57Bl/6 background and age- and sex matched when used in experiments.

### Harvest of primary cells for genetic and functional characterization of *LysM-Myd88^−/−^* and *Tie2-Myd88^−/−^* mice

Peritoneal lavage was performed with 5 ml sterile PBS under isoflurane anesthesia and lavage fluid was collected in PBS containing a final concentration of 10% FBS, 1% antibiotics (penicillin- streptomycin- amphotericin B (Gibco, Paisley, United Kingdom); heart puncture was performed and blood was collected in EDTA or heparin containing tubes; BAL was performed with 10 ml PBS in portions to obtain alveolar macrophages and spleens were harvested. For whole blood stimulation, 100 µl of heparinized blood was pipetted in a 96 wells U-bottom cell culture plate (Greiner bio-one, Alphen a/d Rijn, Netherlands). Spleens were crushed through a 40 µm mesh and after lysis of erythrocytes with an ammoniumchloride containing lysis buffer, splenocytes were seeded in RPMI complete (containing 10% FBS, 1% antibiotics, 10 mM L-glutamine, Gibco) at a density of 500.000 cells per well in 96 wells U-bottom culture plate (Greiner bio-one). Peritoneal and alveolar macrophages were seeded in flat bottom 96 wells cell culture plates (Greiner Bio-one) at a density of approximately 50.000 and 30.000 respectively per well in RPMI complete and left to adhere overnight. Cells were stimulated for 20 hours with the indicated concentrations of heat-killed *K. pneumoniae* or LPS derived from *Klebsiella pneumoniae* (Sigma) diluted in RPMI complete medium in a final volume of 200 microliter.

Whole blood leukocyte genomic DNA was isolated from fresh EDTA blood and primary cells using the Nucleospin Blood Kit (Machery Nagel, Düren, Germany) and in addition, from FACS purified monocytes, neutrophils and lymphocytes. For this, erythrocyte lysis of EDTA blood with ammoniumchloride containing lysis buffer was performed and cells were stained for cell surface molecules using FITC-conjugated anti-mouse Ly6-C &Ly6-G (Gr-1), PE-conjugated anti-mouse CD11b (BD Biosciences) and biotinylated anti-mouse CD115 (eBioscience), secondary staining was performed with streptavidin-APC (BD Biosciences). Monocytes were identified as Gr-1^dim^/CD-115^+^ and neutrophils as Gr-1^high^/Cd115^−^ within the fraction of CD11b^+^ cells, the fraction of Cd11b^−^ cells with a low Side Scatter and Forward Scatter pattern were identified as lymphoid cells [64].

### Real-time PCR

Total RNA was reverse transcribed using oligo (dT) primer and Moloney murine leukemia virus reverse transcriptase (Invitrogen, Breda, The Netherlands).

We quantified the residual amount of the “floxed” region of MyD88 in LysM-*Myd88^−/−^* and Tie2-*Myd88^−/−^* mice in blood and particular cell types using the primer sequences 5′-ACGCCGGAACTTTTCGAT-3′ (forward); 5′-TTTTCTCAATTAGCTCGCTGG-3′ relative to the unaffected *Socs-3* gene with primer sequences 5′- ACCTTTCTTATCCGCGACAG- 3′ (forward) and 5′- TGCACCAGCTTGAGTACACAG-3′ (reverse) in a SybrGreen reaction on an LightCycler system (LC480, Roche Applied Science, Mannheim, Germany). The amount of remaining “floxed” MyD88 region in LysM-MyD88^−/−^ and Tie2-MyD88^−/−^ mice was calculated using the 2^-deltaCt^ (ΔΔCt) method using the amount of genomic DNA from *Myd88^fl/fl^* mice for the no-deletion control [21]. The deletion efficiency was calculated as (1 - residual Myd88^fl^) ×100.

### Induction of pneumonia and sampling of organs

Pneumonia was induced by intranasal inoculation with ∼6×10^3^ colony forming units (CFU) of *K. pneumoniae* serotype 2 (ATCC 43816; American Type Culture Collection, Manassas, VA) and survival was monitored or in separate experiments mice were euthanized after 6 or 24 hours of infection when organs were harvested and processed exactly as described [12], [32].

### Measurements of inflammatory proteins and clinical chemistry

Lung (and cell supernatant) levels of IL-1β, TNF-α, IL-6, IL-10, CXCL-1 and CXCL-2 were measured by ELISA (R&D Systems, Minneapolis, MN). Plasma levels of TNF-α, IL-6, and IL-10 were measured by using a cytometric bead array multiplex assay (BD Biosciences). MPO was measured by ELISA from HyCult Biotechnology (Uden, the Netherlands).Lactate dehydrogenase (LDH) and aspartate aminotransferase (AST were measured using kits from Sigma and a Hittachi analyzer (Boehringer Mannheim).

### Histopathology

Histologic examination of lungs was performed exactly as described [32]. For granulocyte immunohistochemic stainings lung tissue slides were deparaffinized and rehydrated. Endogenous peroxidase activity was quenched by a solution of 0.3% H_2_O_2_ (Merck). Slides were then digested by a solution of pepsin 0.025% (Sigma, St. Louis, MO, USA) in 0.1 M HCl. After being rinsed, the sections were incubated in Ultra V Block (Thermo Scientific, Fremont, CA) and then exposed to a FITC-labeled anti-mouse Ly6-G and Ly6-C monoclonal antibody (BD Pharmingen, San Diego, CA). After washes, slides were incubated with a rabbit anti-FITC antibody (Nuclilab, Ede, The Netherlands) followed by further incubation with Brightvision poly-horseradish peroxidase anti Rabbit IgG (Immunologic, Duiven, The Netherlands), rinsed again and developed using Bright DAB (Immunologic, Duiven, the Netherlands). The sections were counterstained with methyl green and mounted in Pertex mounting medium (Histolab, Gothenburg, Sweden). The Ly-6G and Ly-6C+ percentage of total lung surface was determined with imageJ software (Rasband, W.S., ImageJ, U. S. National Institutes of Health, Bethesda, Maryland, USA, http://rsb.info.nih.gov/ij/, 1997–2011).

### Bone marrow transplantation

BM transplantation was done as described previously [12]. Three groups were generated: Tie2-*Myd88^−/−^* (recipient) +control BM (donor), Tie2-*Myd88^−/−^*+Tie2-*Myd88^−/−^* BM and control+control BM mice. *Myd88^fl/fl^* mice and BM were used as control.

### Statistical analysis

Data are expressed as box-and-whisker diagrams depicting the smallest observation, lower quartile, median, upper quartile, and largest observation (in vivo experiments) or as means ± standard error of the mean (tables, cell stimulation experiments); Comparison of these data was done by Mann Whitney U test. Differences in the proportion of positive cultures were analyzed by Fisher's exact test. Survival curves are depicted as Kaplan-Meier plots and compared using log-rank test. These analyses were done using GraphPad Prism (San Diego, CA). *P*<0.05 was considered statistically significant.

## Supporting Information

Figure S1 **Lung endothelial cell activation as reflected by e-selectin is higher in Tie2-MyD88^−/−^ mice.** Control, LysM-MyD88^−/−^ and Tie2-MyD88^−/−^ mice were inoculated with ∼6×10^3^ CFU *K. pneumoniae* and sacrificed 24 hours later. Homogenates were prepared from right lungs. E-selectin levels are presented in pg/ml lung homogenate **(A)** or plasma **(B)**. Data are mean (SE) of 5–8 mice per group. *p<0.05 vs control mice.
(TIF)

Figure S2 **Absence of hematopoietic or endothelial Myd88 does not impact on organ injury.** Control, LysM-MyD88^−/−^ and Tie2-MyD88^−/−^ mice were inoculated with ∼6×10^3^ CFU *K. pneumoniae* and sacrificed 24 hours later. Plasma levels of LDH **(A)** and AST **(B)**; after 24 hours. Data are expressed as box-and-whisker diagrams depicting the smallest observation, lower quartile, median, upper quartile, and largest observation.
(TIF)

Figure S3 **Local inflammatory response is not affected by the absence of MyD88 expression in the endothelial compartment.** Control and Tie2-MyD88^−/−^ mice were irradiated and injected with control or Tie2-MyD88^−/−^ bone marrow cells. Six weeks after transplantation, mice were infected with 6×10^3^ CFU *K. pneumoniae*. Histological scores 24 hours after infection were determined of control mice transplanted with control bone marrow (Co+ Co BM, grey bars, n = 8) and Tie2-MyD88^−/−^ mice transplanted with control bone marrow (Tie2-MyD88^−/−^+Co BM, white dotted bars) or Tie2-MyD88^−/−^ bone marrow (Tie2-MyD88^−/−^+Tie2-MyD88^−/−^ BM, white bars) **(A)**. Panel **(B)** show representative lung histology of Co+ Co BM mice, Tie2-MyD88^−/−^+Co BM mice and Tie2-MyD88^−/−^+Tie2-MyD88^−/−^ BM, H&E staining, original magnification 20×. Neutrophil influx as reflected by Ly6-G and Ly6-C lung surface positivity **(C)** and whole lung MPO levels **(D)**. Panels **E** shows representative images of Ly-6G and Ly-6C staining on lung slides Co+ Co BM mice, Tie2-MyD88^−/−^+Co BM mice and Tie2-MyD88^−/−^+Tie2-MyD88^−/−^ BM; Data are expressed as box-and-whisker diagrams depicting the smallest observation, lower quartile, median, upper quartile, and largest observation. * *p*<0.05, ** *p*<0.01, *** *p*<0.001 compared to control mice determined with Mann-Whitney *U* test.
(TIF)

Figure S4 **Systemic inflammation is not affected by the absence of MyD88 expression in the endothelial compartment, but lung e-selectin is higher.** Control and Tie2-MyD88^−/−^ mice were irradiated and injected with control or Tie2-MyD88^−/−^ bone marrow cells. Six weeks after transplantation, mice were infected with 6×10^3^ CFU *K. pneumoniae* and sacrificed 24 hours later. Homogenates were prepared from right lungs. E-selectin levels of (Co+ Co BM, grey bars, n = 8) and Tie2-MyD88^−/−^ mice transplanted with control bone marrow (Tie2-MyD88^−/−^+Co BM, white dotted bars) or Tie2-MyD88^−/−^ bone marrow (Tie2-MyD88^−/−^+Tie2-MyD88^−/−^ BM, white bars) are presented in pg/ml lung homogenate **(A)** or plasma **(B)**. Plasma levels of LDH **(C)** and AST **(D)** after 24 hours. Data are expressed as box-and-whisker diagrams depicting the smallest observation, lower quartile, median, upper quartile, and largest observation. *p<0.05 vs control mice.
(TIF)

Table S1 **Inflammatory response in** ***LysM-MyD88^−/−^*** **and** ***Tie2-MyD88^−/−^*** **during** ***K. pneumonia*** **pulmonary tract infection.** Control and Tie2-MyD88^−/−^ mice were irradiated and injected with control or Tie2-MyD88^−/−^ bone marrow cells. Six weeks after transplantation, mice were infected with 6×10^3^ CFU *K. pneumoniae* and sacrificed after 24 hours. Homogenates were prepared from right lungs. Cytokine and chemokine levels are presented in pg/ml lung homogenate or plasma. Data are mean (SE) of 5–8 mice per group. *p<0.05, ** *p*<0.01 vs control mice transplanted with control bone marrow.
(DOCX)
