# Supplementary material for: Hematopoietic but Not Endothelial Cell MyD88 Contributes to Host Defense during Gram-negative Pneumonia Derived Sepsis
Source: PLoS Pathog. 2014 Sep 25;10(9):e1004368. doi: 10.1371/journal.ppat.1004368 (PMC4177915; doi:10.1371/journal.ppat.1004368)
Supplement: Table S1 — Inflammatory response in LysM-MyD88−/− and Tie2-MyD88−/− during K. pneumonia pulmonary tract infection. Control and Tie2-MyD88−/− mice were irradiated and injected with control or Tie2-MyD88−/− bone marrow cells. Six weeks after transplantation, mice were infected with 6×10^3^ CFU K. pneumoniae and sacrificed after 24 hours. Homogenates were prepared from right lungs. Cytokine and chemokine levels are presented in pg/ml lung homogenate or plasma. Data are mean (SE) of 5–8 mice per group. *p<0.05, ** p<0.01 vs control mice transplanted with control bone marrow. (DOCX) [file ppat.1004368.s005.docx]

**Table S1: Inflammatory response in *LysM-MyD88^-/-^* and *Tie2-MyD88^-/-^* during *K. pneumonia* pulmonary tract infection**

| **Recipient** | **Co** | ***Tie2-MyD88^-/-^*** | ***Tie2-MyD88^-/-^*** |
| --- | --- | --- | --- |
| **Bonemarrow** | **Co** | **Co** | ***Tie2-MyD88^-/-^*** |
| ***Lung*** |  |  |  |
| **TNF-α** | 1122 (37) | 1138 (58) | 958 (55) |
| **IL-1β** | 1912 (691) | 3991 (1021) | 2091 (435) |
| **IL-6** | 3697 (1336) | 5142 (1403) | 6222 (694) |
| **IL-10** | 47 (3) | 30 (4)* | 35 (5) |
| **CXCL-1** | 4358 (1194) | 7580 (2371) | 11340 (2560)* |
| **CXCL-2** | 18487 (2479) | 26592 (5357) | 22539 (1449) |
| ***Plasma*** |  |  |  |
| **TNF-α** | 10 (3) | 102 (30) | 136 (85)** |
| **IL-6** | 814 (565) | 2110 (1248) | 3380 (1278)** |
| **IL-10** | bd | bd | bd |
| **IL-12** | bd | bd | bd |
| **CCL-2** | 311 (149) | 3085 (1793) | 3771 (1061)** |
